# Parental Motivation for Introducing Babies’ First Foods and Common Food Allergens

**DOI:** 10.3390/nu17111812

**Published:** 2025-05-27

**Authors:** Zoe Harbottle, Elly Malm Nilsson, Carina Venter, Michael A. Golding, Sandra Ekström, Jennifer L. P. Protudjer

**Affiliations:** 1 Children’s Hospital Research Institute of Manitoba, Winnipeg, MB R3E 3P4, Canada; zoe.harbottle13@gmail.com (Z.H.); michael.golding@umanitoba.ca (M.A.G.); 2 Department of Pediatrics and Child Health, University of Manitoba, Winnipeg, MB R3A 1S1, Canada; 3 Institute of Environmental Epidemiology, Karolinska Institutet, 171 77 Stockholm, Sweden; elly.malm.nilsson@stud.ki.se (E.M.N.); sandra.ekstrom@ki.se (S.E.); 4 Section of Allergy and Clinical Immunology, Children’s Hospital Colorado, University of Colorado, Boulder, CO 80309, USA; carina.venter@childrenscolorado.org; 5 Children’s Hospital Colorado, Denver, CO 80045, USA; 6 Center for Occupational and Environmental Medicine, Region Stockholm, 113 65 Stockholm, Sweden

**Keywords:** early introduction, food allergy, infant feeding, pediatrics

## Abstract

Background/Objectives: Findings from the Learning Early About Peanut trial prompted a shift in clinical practice guidelines to support the early and continuous introduction of allergenic foods to reduce the risk of food allergy. Our study aimed to describe the reasoning behind parents’ decisions on the introduction of first foods to their infants and the age at which parents first introduced common allergens. Methods: Parents of a child aged <18 years old with ≥1 food allergy, who lived in either Canada or the United States, were recruited via social media between March 2021 and February 2022 to participate in an online, anonymous survey. Data were analyzed descriptively and using binary logistic regression. Results: A total of 42 parents completed the survey, the majority being mothers (40/42; 95.2%). Children were, on average, 6.9 ± 0.7 years old. In total, 47.6% of parents introduced first foods between ages 4–5 months, whereas 52.4% introduced first foods at 6 months or older. Cereals were the most frequently introduced first food (54.8%; 23/42). Most parents (71.9%) selected first foods to introduce based on guidance from healthcare providers. Conclusions: For many parents, guidance from healthcare providers is the most influential factor in determining when and what to introduce as first foods to their infant. Although the paradigm shift in infant feeding practices has been well accepted by healthcare providers, this information has not been adequately translated to the general population. This emphasizes the need for healthcare providers to educate and reinforce the importance of early introduction to reduce the risk of food allergy.

## 1. Introduction

From the late 1970s until the early part of the 21st century, the delayed introduction of allergenic foods to infants at or beyond the first year of life was encouraged, in an attempt to reduce the risk of food allergy [1]. Yet, the prevalence of food allergies increased substantially over a similar timeframe [2]. In light of this, Du Toit and colleagues launched the Learning Early About Peanut (LEAP) trial, which sought to determine whether early introduction or avoidance (i.e., the standard of care at the time) was more effective in preventing peanut allergy [3]. Findings from this randomized controlled trial demonstrated that children who were introduced to peanuts early (i.e., within the first year of life) and continued to consume peanuts up to 5 years thereafter were significantly less likely to develop peanut allergy by 5 years of age compared to those who avoided peanuts [3].

LEAP findings led to a paradigm shift in food allergy prevention. Allergy and pediatric societies across the world, including Canada [4,5] and the United States (US) [6], now recommend the introduction of peanut and all other common allergens by around 4–6 months of age or when the infant is developmentally ready. Advice also suggested against delaying the introduction of other allergenic foods. While this leap forward was globally incorporated in revised guidelines, it remains unclear if and how parents have since incorporated these guidelines into their infants’ feeding. To understand this, it is first important to understand why parents make the decisions they do when deciding to first introduce their infants to foods. Our study aimed to describe reasons for parents’ decisions regarding the introduction of first foods to their infants. Our secondary aim was to identify at what age parents introduced first foods and common allergens to their children.

## 2. Materials and Methods

We performed an online, anonymous survey between March 2021 and February 2022 in Canada and the US. Parents of children <18 years old with ≥1 food allergy, were eligible to participate. Food allergy was defined as a convincing history of food allergy, including having an epinephrine autoinjector prescription and parental report of having previously been diagnosed with food allergy by a healthcare provider. Those with an unconvincing history of food allergy (i.e., no epinephrine autoinjector prescription or no prior diagnosis by a healthcare provider) were excluded following the completion of demographic questions if their responses did not meet our definition of food allergy. All participants were recruited via social media. Data were collected on socio-demographics, food allergy characteristics, and dietary intake with an adapted food frequency questionnaire (FFQ) [7]. The adapted FFQ included additional questions related to the timing of solid food introduction in infancy, the type of food introduced, when common allergens were introduced, motivations behind introduction, and intake of foods traditional to a participant’s cultural background. Specific food allergens that were asked about in the adapted FFQ were eggs, peanuts, legumes, and fish. Consent was obtained from all parents. This study was approved by the University of Manitoba Health Research Ethics Board (HS24604), originally approved on 17 January 2021.

Outcome variables were motivation for early introduction (instructions from healthcare providers vs. other causes of motivation); age of introduction of first foods; parental age (≤34 years or >34 years); and presence of chronic conditions, including any allergic conditions (asthma, food allergy, atopic dermatitis, rhinitis/hay fever). Infants’ years of birth were categorized as born before 2016 vs. 2016 onward, which roughly aligned with the LEAP publication [3]. Data were described (n/N, %, mean ± standard deviation [SD]) and analyzed using binary logistic regression, and reported as odds ratios (OR) and 95 percent confidence intervals (95%CIs). We also considered a partially adjusted OR (Model 1; adjusted for parental age, country of residence, and highest level of education) and fully adjusted OR (Model 2; adjusted for parental age, country of residence, highest level of education, annual household income for 2019, and number of people in the household). Food-specific analyses by year of birth (before 2016 vs. 2016 onward) were performed using Fisher’s Exact Tests, as the observations for introduction at age 4–5 months were <5 per age group. Data were analyzed using Stata (Version 18, College Station, TX, USA).

## 3. Results

Our sample (n = 42) was primarily mothers (40/42; 95.2%). Children were, on average, 6.9 ± 0.7 years old, with a relatively even distribution of boys and girls (54.8% and 45.2%, respectively). Amongst children, peanut and tree nut allergies were most common, at 61.9% (26/42) and 52.4% (22/42), respectively, and approximately half were born before 2016 vs. 2016 onward (40.5% vs. 59.5%, respectively) (see Table 1 for participant demographics). Regarding the timing of introduction of first foods, 47.6% of the responding parents introduced first foods at age 4–5 months, and 52.4% introduced first foods at 6 months or older.

**Figure 1:**
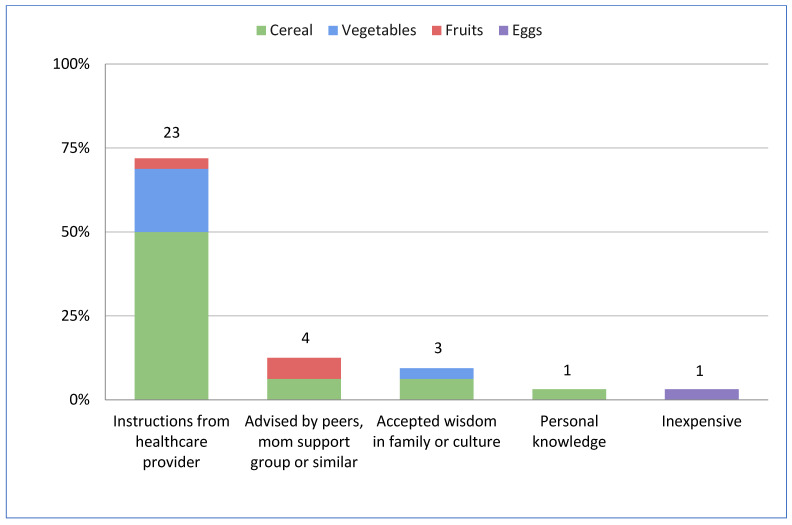
Motivation for introduction of first foods by first food choice (N = 32). Reasoning behind why parents chose the first foods they did when first introducing their infants to foods. Abbreviations: OR, odds ratio; Ref, reference category; 95%CI, 95% confidence interval.

The most frequently introduced first food was cereals (54.8%; 23/42), followed by vegetables (28.6%; 12/42), and then other foods such as eggs and fruits (7/42; 16.7%) (Table 2). The choice of the first food was associated with the country of residence. Compared to Canadians (n = 19), Americans (n = 16) were more likely to introduce vegetables vs. cereals as first food in both the unadjusted (OR 6.86; 95%CI 1.41–33.3; *p* = 0.02) and the fully adjusted (OR 9.62; 95%CI 1.10–84.01; *p* = 0.04) model (Table 2). Other foods, including eggs and fruits, were not included within these analyses as the number of parents choosing these as their infants’ first food was limited. When deciding which food to introduce as the first food to their children, most parents (71.9%) considered instructions from healthcare providers as the most influential factor, followed by advice from peers, mom support groups, or a similar source (12.5%) (Figure 1).

No statistically significant associations between the motivation for the introduction of first foods and parental characteristics were identified (Supplementary Table S1). Considering the associations between the age of introduction of first foods, in an unadjusted analysis, compared to parents age ≤34 years, parents aged >34 years tended to be more likely to introduce first foods between 4 and 5 months of age (OR 2.08; 95%CI 0.55–7.69; *p* = 0.28). The point estimate for the fully adjusted model also provided evidence that parents aged >34 years were more likely to introduce first foods at an earlier age, compared to parents ≤34 years. However, on account of the very wide 95%CI for the fully adjusted model (range >100), we have not reported these data (Supplementary Table S2). Considering the age of introduction of first foods and infants’ year of birth, in both the unadjusted model and the adjusted model, those born in 2016 (the year after LEAP study was published) or later were less likely to be introduced to first foods between 4 and 5 months, but this difference did not reach statistical significance (unadjusted model; OR 0.31; 95%CI 0.55–1.11; *p* = 0.07; fully adjusted model; OR 0.36; 95%CI 0.05–2.70; *p* = 0.32) (Supplementary Table S3). When investigating associations between the timing of introduction of common allergens and infants’ year of birth, there was no difference in age of introduction for either peanuts (*p* = 0.13) or eggs (*p* = 0.63) between those born prior to 2016 vs. 2016 or later. Owing to a few observations of introduction prior to 2016, logistic regression analyses were not possible.

This study provides evidence that most parents select cereals (wheat, oat, or rice-based) as first foods, which is influenced by instructions from healthcare providers. Although the findings regarding introduction at ages 4–5 months vs. 6+ months did not significantly differ between parents of infants who were born before 2016 vs. 2016 onward, the direction of the relationship is not supportive of earlier introduction after the LEAP publication. Older parental age (>34 years) was associated with an increased odds of earlier first food introduction; however, this relationship did not quite reach statistical significance (*p* = 0.06).

## 4. Discussion

The main challenges associated with the early introduction of foods are fear of allergic reactions, lack of patient education, poor access to health services, and poor child cooperation [8]. The paradigm shift in infant feeding guidelines, which resulted from the LEAP findings, has been adopted by healthcare providers in some regions; however, the level of implementation varies globally [9,10]. Our findings provide modest evidence that these instructions have not been integrated amongst the general population. Consequently, it behooves healthcare providers to continue to reinforce the need for early introduction, by ages 4–6 months, or when the infant is developmentally ready, to reduce the risk of food allergy. While American vs. Canadian parents were significantly more likely to introduce vegetables as a first food, American and Canadian guidelines recommend both infant cereals and vegetables as first foods [11,12].

## 5. Conclusions

While our findings provide valuable information on the implementation of early introduction guidelines among Canadian and American parents, there are some limitations that should be discussed. Our study was based on retrospective data; it is possible that parents of children, particularly those who were older, may not clearly remember the order of introduction of foods [13]. As well, the small sample size, which was composed primarily of mothers, and the lack of data on cultural background may limit the generalizability of the findings. As parents value guidance from healthcare providers above other recommendations, healthcare providers must remain committed to counseling new parents on the evidence-based importance of early introduction.

## Figures and Tables

**Table 1:** Socio-demographics of participating dyads (N = 42).

Participant Characteristics		
	n	%
**Characteristics of caregiver**		
**Respondents’ relationship to child**		
Mother	40	95.2
Father	2	4.8
**Age (years)**		
≤34	14	33.3
>34	28	66.7
**Country of residence**		
United States	19	45.2
Canada	23	54.8
**Highest level of education (n = 39)**		
High school, college, trade school, undergraduate degree	18	46.2
Graduate or professional degree	21	53.8
**Chronic conditions (N = 42)**		
Yes; any	20	47.6
Yes; allergic chronic conditions (i.e., asthma, food allergy, atopic dermatitis, rhinitis/hay fever) (N = 20)	13	65
Characteristics of child *		
**Year of birth**		
Before 2016	17	40.5
2016 and onwards	25	59.5
**Sex**		
Male	23	54.8
Female	19	45.2
**Age at diagnosis of food allergy (years) (n = 41)**		
0–2	37	90.2
≥3	4	9.8
Types of food allergies **		
Peanut	26	61.9
Milk	24	57.1
Tree nuts	22	52.4
Egg	14	33.3
Sesame	8	19
Soy	5	11.9
Wheat or triticale	5	11.9
Fish	3	7.1
Crustaceans or mollusks (shellfish)	2	4.8
**Characteristics of households**		
**Annual household income 2019 in Canadian dollars (CAD) (n = 39)**		
Less than CAD 100,000	17	43.6
More than CAD 100,000	22	56.4
**Number of people in household**		
3 February	14	33.3
6 April	28	66.7
**Number of children in household**		
1	13	30.9
≥2	29	69.1

* data reported for the eldest child with food allergy from each family; ** not mutually exclusive.

**Table 2:** Associations between the introduction of first foods, vegetables (N = 12) versus cereals (N = 23), by caregiver characteristics.

		Unadjusted			Model 1 *			Model 2 **		
	n	OR	95%CI	*p*-Value	OR	95%CI	*p*-Value	OR	95%CI	*p*-Value
**Age of caregiver (years)**										
≤34	9	Ref			Ref			Ref		
>34	26	2.19	0.38–12.70	0.38	1.59	0.22–11.36	0.65	1.03	0.07–15.14	0.99
**Country of residence**										
Canada	19	Ref			Ref			Ref		
United States	16	**6.86**	**1.41–33.29**	**0.02**	**7.37**	**1.13–48.13**	**0.04**	**9.62**	**1.10–84.01**	**0.04**
**Highest level of education of caregiver**										
Undergraduate degree or less	14	Ref			Ref			Ref		
Graduate or professional degree	19	1.05	0.25–4.42	0.95	0.44	0.07–2.73	0.38	0.72	0.10–5.16	0.75

* adjusted for age of caregiver, country of residence, and highest level of education. ** adjusted for age of caregiver, country of residence, highest level of education, annual household income 2019, and number of people in household. Abbreviations: OR, odds ratio; Ref, reference category; 95%CI, 95% confidence interval.

## Data Availability

The data presented in this study are available on request from the corresponding author due to the nature of the data and small sample sizes.

## Supplementary Materials

The following supporting information can be downloaded at https://www.mdpi.com/article/10.3390/nu17111812/s1, Table S1: Associations between the motivation for introduction of first foods, instructions from care provider (N = 23) versus other causes of motivation (N = 9), by caregiver characteristics *; Table S2: Associations between the age of introduction of first foods, 6 months or older (N = 22) versus age 4-5 months (N = 20), by caregiver characteristics; Table S3: Associations between the age of introduction of first foods, 6 months or older (N = 22) versus age 4-5 months (N = 20), by child’s year of birth adjusted for caregiver characteristics.

## Abbreviations

FFQ	Food frequency questionnaire
LEAP	Learning Early About Peanut
OR	Odds ratio
SD	Standard deviation
US	United States of America
95%CI	95 percent confidence interval
