# Supplementary material for: Parental Motivation for Introducing Babies’ First Foods and Common Food Allergens
Source: Nutrients. 2025 May 27;17(11):1812. doi: 10.3390/nu17111812 (PMC12158072; doi:10.3390/nu17111812)
Supplement: Supplementary file 1 [file nutrients-17-01812-s001.zip › nutrients-3621293-supplementary.pdf]

Supplementary Table S1. Associations between the motivation for introduction of first foods, instructions from care provider (N=23) versus other causes of motivation (N=9), by caregiver characteristics*

		Unadjusted			Model 1**			Model 2***		
	n	OR	95%CI	p-value	OR	95%CI	p-value	OR	95%CI	p-value
**Age of caregiver (years)**										
≤ 34	10	Ref			Ref			Ref		
> 34	22	0.44	0.09 - 2.21	0.32	0.29	0.04 - 1.93	0.20	0.27	0.02 - 4.26	0.36
**Country of residence**										
Canada	18	Ref			Ref			Ref		
United States	14	1.04	0.22 - 4.91	0.96	0.95	0.17 - 5.13	0.95	0.59	0.09 - 3.99	0.59
**Highest level of education of caregiver**										
High school, college, trade school, undergraduate degree	14	Ref			Ref			Ref		
Graduate or professional degree	17	2	0.40 - 10.09	0.40	3.34	0.50 - 23.19	0.22	3.40	0.37 - 30.88	0.28

*category of other causes of motivation include; advised by peers, parent support group or similar, accepted wisdom in family or culture, personal knowledge and cost.
**adjusted for age of caregiver, country of residence and highest level of education
***adjusted for age of caregiver, country of residence, highest level of education, annual household income 2019 and number of people in household
Abbreviations: OR odds ratio; Ref Reference category; 95%CI 95% percent confidence interval

Supplementary Table S2. Associations between the age of introduction of first foods, 6 months or older (N=22) versus age 4-5 months (N=20), by caregiver characteristics.

		Unadjusted			Model 1*			Model 2**		
	n	OR	95%CI	p-value	OR	95%CI	p-value	OR	95%CI	p-value
**Age of caregiver (years)**										
≤ 34	14	Ref			Ref			Ref		
> 34	28	0.48	0.13 - 1.81	0.28	0.26	0.05 - 1.39	0.115	0.12	0.01 - 1.08	0.06
**Country of residence**										
Canada	23	Ref			Ref			Ref		
United States	19	0.69	0.20 - 2.35	0.56	0.60	0.15 - 2.43	0.47	0.45	0.08 - 2.48	0.36
**Highest level of education of caregiver**										
High school, college, trade school, undergraduate degree	18	Ref			Ref			Ref		
Graduate or professional degree	21	1.33	0.38 - 4.73	0.66	2.81	0.57 - 13.84	0.20	4.63	0.74 - 29.13	0.10

*adjusted for age of caregiver, country of residence and highest level of education

**adjusted for age of caregiver, country of residence, highest level of education, annual household income 2019 and number of people in household

Abbreviations: OR odds ratio; Ref Reference category; 95%CI 95% percent confidence interval

Supplementary Table S3. Associations between the age of introduction of first foods, 6 months or older (N=22) versus age 4-5 months (N=20), by child's year of birth adjusted for caregiver characteristics.

		Unadjusted			Model 1*			Model 2**		
	n	OR	95%CI	p-value	OR	95%CI	p-value	OR	95%CI	p-value
**Year of birth**										
Children born before 2016	25	Ref			Ref			Ref		
Children born after 2016	17	3.26	0.90-11.81	0.07	2.27	0.43 - 12.05	0.34	2.78	0.37 - 20.80	0.32

*adjusted for child's year of birth, age of caregiver, country of residence and highest level of education

**adjusted for child's year of birth, age of caregiver, country of residence, highest level of education, annual household income 2019 and number of people in household

Abbreviations: OR odds ratio; Ref Reference category; 95%CI 95% percent confidence interval
